# Seeing the Tree of Life behind the phylogenetic forest

**DOI:** 10.1186/1741-7007-11-46

**Published:** 2013-04-15

**Authors:** Pere Puigbò, Yuri I Wolf, Eugene V Koonin

**Affiliations:** 1 National Center for Biotechnology Information, National Library of Medicine, National Institutes of Health, Bethesda, MD 20894, USA

## 

In the article entitled 'Search for a Tree of Life in the thicket of the phylogenetic forest', published in 2009 in *Journal of Biology *[1] (see also the accompanying comment [2]), we presented evidence that the traditional Tree of Life (TOL) can and should be replaced with a statistical central trend in the genome-wide compendium of phylogenetic trees that reflects the coherence between the evolutionary histories of different genes and was later denoted the Statistical Tree Of Life (STOL) [3]. Since Darwin's day, the TOL is the dominant icon of evolutionary biology [4,5], the basis of taxonomy and an essential framework for evolutionary reconstructions. In the late 1970s, ribosomal (r)RNA was introduced as a universal phylogenetic marker, primarily through the work of Carl Woese and colleagues [6,7], and the rRNA tree, complemented with trees for other universal genes such as the large RNA polymerase subunits, became the standard model for TOL study.

Technical difficulties notwithstanding, progress in genome sequencing combined with advances in phylogenetic analysis seemed to put a well-resolved TOL within reach [8,9]. However, as soon as a reasonable number of complete genome sequences of bacteria and archaea became available, phylogenomics - genome-wide phylogenetic analysis of individual gene trees - hopelessly marred this neat picture by showing that the trees of different genes generally had different topologies. The topological inconsistencies between gene trees were far too extensive to be dismissed as phylogenetic artifacts, leading to the realization that no single gene tree, including those for universal genes such as rRNA, could represent the evolution of genomes in its entirety. Hence the concepts of horizontal genomics or a 'net of life' were brought about to replace the simple notion of the TOL [10,11]. In the extreme, several influential studies proposed to dispense with 'tree thinking' altogether as an artificial construct having little to do with actual evolution, at least as far as bacteria and archaea are concerned [12-15].

The concept of 'horizontal genomics' involves an internal contradiction because the notion of horizontal gene transfer (HGT) inherently implies the existence of a standard of vertical, tree-like evolution, and most of the existing methods for HGT detection are based on the comparison of gene trees to a standard 'species tree', in practice often the rRNA tree [16,17]. If the vertical standard does not exist, the concept of HGT becomes effectively meaningless, so all we can talk about is a network of life, with nodes corresponding to genomes and edges reflecting gene exchange [18]. The stakes here are high because replacement of the TOL with a network graph would change our entire perception of the process of evolution and invalidate all evolutionary reconstruction based on a species tree. However, the tree representation is by no means superfluous to the description of evolution because the very process of the replication of genetic information implies a bifurcating graph - in other words, a tree [19]. Thus, the key question is [1,20]: in the genome-wide compendium of phylogenetic trees, that we denoted the Forest Of Life (FOL), can we detect any order, any preferred tree topology (branching order) that would reflect a consensus of the topologies of other trees?

We set out to address the above question as objectively as possible, first of all dispensing with any pre-selected standard of tree-like evolution. The analyzed FOL consisted of 6,901 maximum likelihood phylogenetic trees that were built for clusters of orthologous genes from a representative set of 100 diverse bacterial and archaeal genomes [1]. The complete matrix of topological distances between these trees was analyzed using the Inconsistency Score, a measure that we defined specifically for this purpose that reflects the average topological (in)consistency of a given tree with the rest of the trees in the FOL (for the details of the methods employed in this analysis, see [21]). Although the FOL includes very few trees with exactly identical topologies, we found that the topologies of the trees were far more congruent than expected by chance. The 102 Nearly Universal Trees (NUTs; that is, the trees for genes that are represented in all or nearly all archaea and bacteria), which include primarily genes for key protein components of the translation and transcription systems, showed particularly high topological similarity to the other trees in the FOL. Although the topologies of the NUTs are not identical, apparently reflecting multiple HGT events, these transfers appeared to be distributed randomly. In other words, there seem to be no prominent 'highways' of HGT that would preferentially connect particular groups of archaea and bacteria. Thus, although the NUTs cannot represent the FOL completely, they appear to reflect a significant central trend, an attractor in the tree space that could be equated with the STOL (Figure 1).

**Figure 1:**
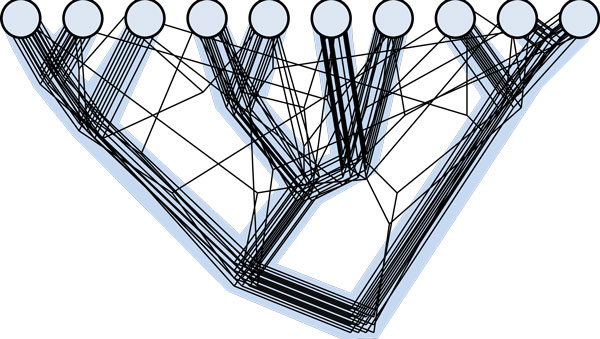
**The central tree-like trend in the phylogenetic forest of life**. The circles show genomes of extant species and the grey tree in the background shows the statistical central trend in the data. For the purpose of illustration, the figure shows an 'FOL' made of 16 trees with 20 deviations from the central tree-like pattern.

The set of 6,901 phylogenetic trees that comprise the FOL has become a launching pad for several new studies addressing various aspects of prokaryote evolution and general questions of evolutionary biology. In our own hands, the sequel to the original FOL study involved quantitative dissection of the evolution of prokaryotes into tree-like and web-like components [22]. We applied the approach known as quartet analysis to quantify the contributions of these two distinct modes of evolution [21] and found that, although diverse routes of net-like evolution collectively dominate the FOL, the pattern of tree-like evolution that reflects the generally consistent topologies of the NUTs is the most prominent coherent trend [22]. Thus, the ubiquity of HGT notwithstanding, this central tree-like trend reflects a major aspect of genome evolution and hence has a legitimate claim to represent the STOL.

Having established the validity of the STOL, we employed it to reassess a fundamental aspect of evolutionary theory, the molecular clock (MC) model under which genes evolve at approximately constant gene-specific rates [23]. Using the supertree of the NUTs as a proxy for the STOL, we compared the fits of approximately 3,000 largest trees (that is, the trees with the largest number of species) from the FOL to the supertree that was constrained either under the MC assumption or according to another, more general model that we denoted Universal PaceMaker (UPM) of genome evolution [24]. Under the UPM model, the rate of evolution changes synchronously across genome-wide sets of genes in each evolving lineage (the genes accelerate or decelerate their evolution in sync), thus explaining the universal distribution of evolutionary rates of orthologous genes from diverse life forms [25,26]. However, unlike the MC model, the UPM model does not assume conservation of absolute gene-specific evolutionary rates. We showed that the UPM model fits the data substantially better than the MC model, with the implication that the MC should be replaced by a more general constraint on the evolutionary process under which only the relative evolutionary rates of the genes are conserved [24,27]. Others have also employed the FOL to test new approaches for tree comparison and 'harvest' different kinds of evolutionary signals, in particular those that reflect HGT between diverse bacteria and archaea with similar life styles [28].

The study of the interplay between the vertical and horizontal trends in the evolution of prokaryotes continues, stimulated by the rapid accumulation of diverse archaeal and bacterial genome sequences. For example, a new and potentially promising twist of this theme is the use of shared HGT events to refine and root the species trees for prokaryotic phyla [29]

A key fact established by comparative genomics is that we already know all the NUTs: no new (nearly) universal genes can possibly be discovered, and it is equally unlikely that a substantial fraction of the NUTs will lose the 'nearly universal' status [30,31]. Thus, given that the coherent topologies of the NUTs seem to adequately represent a central statistical trend in the FOL, the STOL appears to be here to stay and could become a solid foundation for a genome-based classification of bacteria and archaea [32], and perhaps even more importantly, a robust framework for evolutionary reconstruction.

## Note

This article is part of the *BMC Biology *tenth anniversary series. Other articles in this series can be found at http://www.biomedcentral.com/bmcbiol/series/tenthanniversary.
